# Recent nutritional strategies and feed additives to stimulate proper rumen development in young goats

**DOI:** 10.1093/tas/txae164

**Published:** 2025-03-08

**Authors:** Mahmoud M Abdelsattar, Wei Zhao, Mohamed Diaby, Einar Vargas-Bello-Pérez, Naifeng Zhang

**Affiliations:** Key Laboratory of Feed Biotechnology of the Ministry of Agriculture and Rural Affairs, Institute of Feed Research of Chinese Academy of Agricultural Sciences, Beijing, China; Department of Animal and Poultry Production, Faculty of Agriculture, South Valley University, Qena, Egypt; Key Laboratory of Feed Biotechnology of the Ministry of Agriculture and Rural Affairs, Institute of Feed Research of Chinese Academy of Agricultural Sciences, Beijing, China; College of Animal Science & Technology, Yangzhou University, Yangzhou, Jiangsu, China; Department of International Development, School of Agriculture, Policy and Development, University of Reading, Reading RG6 6EU, UK; Facultad de Zootecnia y Ecología, Universidad Autónoma de Chihuahua, Chihuahua, Mexico; Key Laboratory of Feed Biotechnology of the Ministry of Agriculture and Rural Affairs, Institute of Feed Research of Chinese Academy of Agricultural Sciences, Beijing, China

**Keywords:** early weaning, goat kids, nutritional regulation, rumen development, volatile fatty acids

## Abstract

Domestic goats (*Capra aegagrus hircus*) are important producers of milk, meat, and hair. The early weaned goats may face fundamental issues related to the incomplete rumen development to deal with the transition from liquid feeds into solid feeds. Therefore, the present review focuses on the nutritional strategies and feeding methods to enhance the proper rumen morphological development, fermentation efficiency and microbiota structure in young goats. The enhanced rumen development caused by these nutritional strategies can have lasting positive effects on their overall growth performance and health status, leading to decreasing mortality rates and susceptibility to disease after weaning. A wide range of areas was summarized including liquid feed management in preweaning goats (colostrum, milk, and milk replacer), solid feed management (concentrate and roughages), endogenous and exogenous volatile fatty acids and ketones, plant extracts, prebiotics and probiotics as well as rumen microbial contents that can be incorporated into the kids as an alternative to antibiotics to avoid pathogens and enhance the proper establishment of microbial community. Such nutritional strategies and current breeding recommendations can be used for the development of young goats’ production systems to enhance the long-term digestive function efficiency in goats.

## Teaser

•Early inclusion of solid feed helps to improve rumen development in goats.

•Plant extracts, probiotics, and microbial inoculants can improve rumen development in goats.

## Introduction

The goat, as it is called the small cow, is considered an important source of meat, milk, hair, and skin production (Mongini, 2013). Recent studies have been focused on strategies to enhance goat welfare, health, and production efficiency. Growth performance, production, and health status are all boosted by the rumen, a crucial digestive organ in ruminants, where a complex community of microorganisms, including bacteria, fungi, and protozoa, work together to break down complex plant materials and convert them into usable nutrients for the animal. The balance of these microbial populations is essential for proper digestion and the overall health of the animal (Zhang et al., 2022). At weaning, the early stage of life is important for the manipulation of rumen morphological and microbial development, as well as the rumen metabolic function. However, the early weaning due to the changes of feeding structure could hurt the digestive system and induces gut microbial imbalance and intestinal injury, leading to growth retardation, inflammation, malabsorption, diarrhea, and mortality (Meale et al., 2016; Wang et al., 2016; Liao et al., 2019; Zhang et al., 2022). At this stage, rumen lacks enough microbial structure, metabolic activity, and epithelium development to adapt to the new solid starter diet. Therefore, the early development of the gastrointestinal tract is critical for rumen microbial colonization and adaptability (Arshad et al., 2021), as well as the smooth transition from liquid to solid meal (Baldwin et al., 2004; Malmuthuge et al., 2013; Rey et al., 2014), can lead to improved nutrient utilization, growth rates and better health that lasts throughout life (De Barbieri et al., 2015).

**Figure 1.**
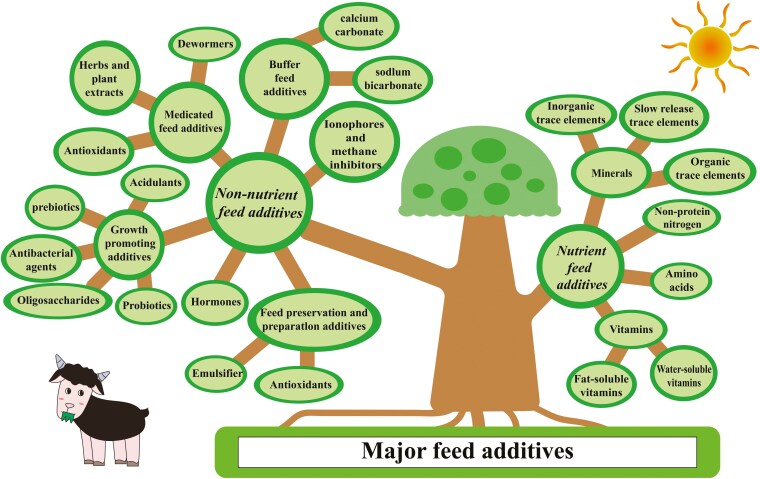
Major feed additives in animal nutrition.

The intake of healthy and high-quality feeds is important in the case of newborn goat kids, as it enhances rumen morphology which allows for more efficient absorption of nutrients. In addition, feeding can promote fermentation process and rumen microbiota towards a more diverse and beneficial microbial population, with key implications for the health, performance, and long-term digestive function of goat kids. After birth, colostrum and milk or milk replacer feeding can have a big impact on kid’s overall performance, health, and survival rates (Zamuner et al., 2023). While the esophageal groove prevents colostrum or milk from entering rumen, solely liquid feeding minimizes rumen fermentation substrates required for rumen development (Khan et al., 2011; Jiao et al., 2015). On the other hand, with the introduction of solid diets, the rumen experiences significant morphological changes (e.g., greater papilla and higher rumen weight) and develops into a functioning organ (Lv et al., 2019; Chai et al., 2021). The fibrous plant polymers are decomposed into volatile fatty acids (**VFAs**) by bacteria, fungus, and protozoa (Jami et al., 2013), which can be used as a good source of energy for rumen growth (McCann et al., 2014). Therefore, the dietary supplementations of the VFA and ketone bodies were reviewed as a core metabolite linked with rumen metabolic development (Lane et al., 2002; Khan et al., 2011). Thus, feeding style, feeding times, and feed composition are important for proper rumen development (Abecia et al., 2014; Sun et al., 2017; Zhang et al., 2019a). Nowadays, many feed additives are being used to improve the feed quality, growth performance, and health of livestock (Fig. 1). Several plant extracts have been used in the goat industry as an alternative to antibiotics and to enhance the feed quality, improve rumen fermentation, and reduce methanogenesis and inflammations (Busquet et al., 2005; Harun et al., 2017; Okoruwa and Ikhimioya, 2020). Another popular antibiotic alternative reviewed here was probiotics or direct-fed ruminal microbiota, according to their role in improving goats’ performance and maintaining healthy rumen against a variety of diseases such as diarrhea (Zhang et al., 2021a; Reuben et al., 2022). In addition, the lack of rumen microbial ecosystem due to the isolation of kids from adult goats with weaning can be minimized by using specific probiotic strains (Abbasi et al., 2018; Shahid et al., 2020; Zhang et al., 2021a), rumen fluid inoculation (De Barbieri et al., 2015; Belanche et al., 2020a; Palma-Hidalgo et al., 2021), and fecal microbiota transplantation techniques (Kim et al., 2021). It has been reported that modulating probiotics intake after weaning may have a promising strategy for gut microbial balance and the prevention of inflammatory diseases in goats (Zhang et al., 2022).

This comprehensive review represents various feeding strategies and feed additives to be used in the early stages of life for the improvement of rumen fermentation and microbial structure to reduce weaning stress and finally promote performance and health status. This review aimed to summarize the recommendations regarding dietary management of rumen development in goats from birth to postweaning, while other studies on manipulating rumen function in growing or adult goats and even other ruminants were included when pertinent to the discussion.

## Materials and Methods

The information search focused on the impact of specific feeds (colostrum, milk, milk replacer, concentrate, and roughage) and feed additives (VFA and ketones), plant extracts, probiotics, and microbial transplants on supporting the development of a healthy rumen environment, and ultimately the overall performance and health status in young goats. Using the databases from PubMed, Science Direct, Web of Science, Scopus, and Google Scholar, the literature search was done until March 2024. Only peer-reviewed English-language studies from recent years about the development of nutritional strategies in controlling weaning stress and boosting rumen development were summarized; contributions from conferences and congresses were not included. Although we tried to concentrate on studies done on young goats, we also included studies done on adult goats or other relevant ruminant species but on a narrow scale. Therefore, there may be nutritional treatments that have proven their effectiveness in other ruminants such as calves or lambs and are not mentioned here.

### Liquid Feeds

At birth, the newborn ruminants have a digestive system with a nonfunctional rumen. The esophageal groove prevents colostrum or milk from entering the rumen, which decreases the rumen fermentation substrates necessary for rumen growth and development (Khan et al., 2011; Jiao et al., 2015). However, goat colostrum at partum is richer in fat, protein, somatic cells (leukocytes and epithelial cells), lactic acid, and immunity molecules (immunoglobulin G, A, and M) than the following milk secretions (Sanchez-Macias et al., 2014).

Thus, colostrum, due to its high nutrient content, growth factors, and immunoglobulins, is considered a natural prebiotic and can positively influence the immunological response and the antioxidant status of newborn mammals during the early stages of life (Martin et al., 2021), which positively impact the gut performance and metabolism and the health situation (Blum, 2006; Ontsouka et al., 2016). Away from the rumen, the newborn morphological development and functional maturity of the gut depend on colostrum feeding (Pyo et al., 2020), by supporting the absorptive capacity of nutrients and nutrient availability (Blum, 2006). Colostrum, as a prebiotic, provides nutrients for host development and the early microbiota community within the digestive tract (Tao et al., 2009; Cabrera-Rubio et al., 2012).

Moreover, the formation of the microbial components for establishing the gastrointestinal tract, the early establishment of gut microbiota, and the promotion of health status in goats are all facilitated by the colostrum microbial communities (Niyazbekova et al., 2020). The high quality of colostrum is based on the rate multiplication of the probiotic microbiome and the reduction of pathogenic bacteria (Ma et al., 2019; Puppel et al., 2020). Colostrum is considered the second stage of the microbiome development of the digestive tract after birth, while the fetal period is the initial stage and the third phase starts after weaning (Zou et al., 2020).

It has also been reported that within the dominated *Proteobacteria* phyla at 0 d age, higher relative abundance was observed for the genus *Escherichia* (Jiao et al., 2015, 2017) or *Mannheimia* (Wang et al., 2017; Zou et al., 2020). After consuming colostrum, some of these bacteria were either quickly eliminated or remained at undetectable levels (Wang et al., 2017; Zou et al., 2020). Then, the delay of colostrum feeding to 6 and 12 h reduced the passive transfer of immunoglobulins leading to about 31% mortality in the neonatal period (Fischer et al., 2018) and the delay in bacterial growth and immune cell development could make small ruminants more susceptible to infection before weaning (Zhang et al., 2021b). Therefore, colostrum may enhance neonatal development due to the enhanced development of the intestine due to the improved nutrient absorption, protein synthesis, and anabolic processes.

In addition to colostrum, goat milk with a distinct chemical, biological, physical, and nutritional profile, as well as a greater microbial diversity, is considered a beneficial probiotic and antibacterial agent that has a positive impact on the immune and the maturation and colonization of gut microbiota (O’Sullivan et al., 2015; Zhang et al., 2017). The maternal milk components such as antibodies, lactoferrin, casein, and oligosaccharides promote the development of probiotic microbes such as *Bifidobacterium* spp. in the digestive system while warding off infections, as reviewed by Reuben et al. (2022). In addition, Maternal milk as a source of microbiota contains several beneficial microbiota such as *Streptococcus*, *Veillonella*, *Gemella*, *Enterococcus*, *Clostridia*, *Bifidobacterium*, *Lactobacillus*, *Sphingomonas*, *Serratia*, *Escherichia*, *Enterobacter*, *Ralstonia*, *Bradyrhizobium*, *Propionibacterium*, *Actinomyces,* and *Corynebacterium* (Hunt et al., 2011; Cabrera-Rubio et al., 2012). Thus, increasing the amount of milk fed to goat kids could boost their live body weight (**BW**) growth (Khatun et al., 2019). However, the beneficial effects of milk depend on the milk quality, which may influence the colonization of microbiota (Han et al., 2020).

Moreover, milk replacer is artificial milk created by substituting non-milk protein for dam’s milk protein (Chapman et al., 2016). Milk replacers can be an economical choice for feeding newborn goats versus whole milk, which reduces their dam’s intake and the empty pregnancy ratio and increases reproductive efficiency (Santos et al., 2018). Thus, it has been reported that early weaning and milk replacer are considered the primary methods to increase the breeding efficiency of ewe and the growth performance of goat kids (Hu et al., 2020), subsequently alleviating the weaning stress (Liao et al., 2021). Milk replacer decreases the morbidity and mortality caused by the early nutrient intake shortage (Emsen et al., 2004). Goat kids offered milk replacer had earlier rumen development, including rumen weight, papilla length and width, papilla density, and rumen wall thickness than those consumed whole milk (Sarker et al., 2015), which might be attributed to the fibrous plant-based particles present in soymilk. In early weaning kids, milk replacers maintained a healthy intestinal microbiota, improved immunity, and decreased stress responses (Wang et al., 2021), regulating their growth performance and rumen development (Bhatt et al., 2009). Moreover, the relative abundance of intestinal pathogens, such as *Clostridium*, decreased in milk-replacer-treated goats, while several beneficial bacteria (*Ruminococcaceae*, *Ruminococcus*, *Christensenellaceae*, and *Ruminiclostridium*), have shown a progressive rise in their numbers (Li et al., 2021a). In addition, the ratio of beneficial to harmful bacteria in the gut microbiome of young goats may be increased as a result of milk replacement feeding (Han et al., 2020). Supplementing with milk replacer may also help for the rumen microbial establishment (Zhang et al., 2019b). The *Ruminococcaceae* NK4A214 group, *Akkermansia*, *Veillonella*, *Anaerovibrio*, and *Ruminococcus* were higher in the milk-replacer-fed goats’ rumen than in the control goats, however *Turicibacter* was lower (Han et al., 2020). Intensified milk replacers can, therefore, trigger a milder immune response that modifies the rumen bacteria and lowers the relative number of harmful bacteria (Zhang et al., 2018a). In contrast, other studies reported that feeding milk replacer leads to disruption in the abundance of *Bacteroides* spp., and daily weight gain in goat kids compared to the dam’s milk (Zhang et al., 2024), indicating that further reevaluation is still warranted to improve the use of milk replacers.

The effects of milk/milk replacer feeding frequency on goat kids’ growth and health are gaining more interest. Bélanger-Naud and Vasseur (2021) suggested that after birth, goat kids should receive 2 to 4 colostrum meals per day to ensure they obtain the necessary antibodies and nutrients. However, Vickery et al. (2023) showed that goat kids may consume up to 6 milk meals per day with high individual differences between the kids. Vickery et al. (2022) reported that most commercial farms (78.3%) feed 3 to 4 meals daily, while 10% feed twice a day. Increasing milk feeding frequency can improve nutrient utilization by reducing meal size and accelerating abomasal emptying (Orellana Rivas et al., 2022). While milk feeding frequency plays a vital role in kid immunity, it also incurs higher labor expenses (Zamuner et al., 2023). Consequently, some farmers consider reducing milk feeding frequency as a strategy to reduce labor costs (Zened et al., 2024). This approach can also encourage suckling animals to explore alternative feed sources, such as starter diets (Jensen et al., 2020; Jafari et al., 2021), which facilitates the transition to solid diets (Zamuner et al., 2023). Studies have indicated that decreasing milk feeding frequency has minimal effects on feed intakes, growth performance, and long-term health in calves (Welk et al., 2023; Revilla-Ruiz et al., 2024), as well as on rumen fermentation (Ahmadi et al., 2022) and the colonization of ruminal bacteria (Zened et al., 2024). Accordingly, about 89.5% of dairy goat farms decrease the daily milk meals after the first week of age (Vickery et al., 2022).

Although liquid feeds could increase the efficiency and rapidity of growth in newborn ruminants; however, consuming only milk makes the digestive system not optimize the digestion of solid feeds (Yanez-Ruiz et al., 2015). Solely feeding newborn goats a liquid diet, such as goat milk, can lead to a lack of solid feed intake, leading to little rumen fermentation, less VFA absorption, and postponed rumen growth (Quigley et al., 2006; Sarker et al., 2015; Abdelsattar et al., 2022b). The rumen microbial structure and composition in starter-fed goats develop more easily and quickly than in milk-fed goats (Jiao et al., 2015). In addition, compared with the early solid diet, encouraging milk replacer feeding, similar to milk feeding, has a detrimental influence by lowering neonate performance, reducing solid feed intake, and easing rumen development (Górka et al., 2011). Thus, goats offered milk replacers as a liquid diet had a rumen with small papillae, resulting in a decreased population of bacteria adherent to the rumen epithelial tissue (Chai et al., 2021). It is important to gradually introduce solid feed into their diet to support proper rumen development and ensure balanced nutrition (Chai et al., 2017). Another potential issue with solely liquid diets is the risk of overfeeding or underfeeding. It can be challenging to accurately measure and monitor the amount of liquid diet consumed by each kid, which may result in nutritional imbalances or digestive disturbances. Meale et al. (2016) reported that although excessive milk intake encourages weight increase, it does not prepare the rumen for proper weaning. Furthermore, some liquid diets may not provide all the essential nutrients and minerals required for optimal growth and development. For example, milk replacers may lack certain vitamins or minerals that are naturally present in goat milk. Inadequate nutrition can result in health problems, stunted growth, and reduced immune function in newborn goats. In addition, the non-milk protein can reduce fat and protein digestibility by altering normal clot formation (Sanz Sampelayo et al., 1990). Although restricted milk feeding promoted goat kids to eat solid feed and, as a result, early weaning (Lu and Potchoiba, 1988; Goetsch et al., 2001). Hence, in addition to other nutritional methods like maximizing feeder placement and kind of solid feed, decreasing milk allowance or step-down weaning procedures have been suggested to increase the solid feed intake prior to weaning (Mikus et al., 2020; Parsons et al., 2020). However, other studies showed that the gradual reduction of milk volume did not motivate kids to consume further pelleted feed (Zobel et al., 2020). In addition, Paez Lama et al. (2014) found nonsignificant differences in dry matter intake (**DMI**) between natural suckling kids and artificially reared kids. For the optimization of weaning strategies, further research is required to improve our knowledge on the impact of colostrum and milk on the gut microbiota and immune system, and the performance of young goats.

### Solid Feeds

Goats rely on dry feed to meet their nutritional needs after weaning. Goats may not be ready for their postweaning diet if they do not consume enough solid feed (such as grain or hay) before weaning (Zobel et al., 2020), After weaning, they had poor growth and were hungry for a long time until solid feed met their needs for maintenance and growth (Costa et al., 2015). As a result, it is critical to start providing solid feed to goat kids at a young age to enhance the formation of the digestive tract microbiota, which is critical for efficient post-ruminal nutrient absorption (Htoo et al., 2018; Malmuthuge et al., 2019). Providing solid feed throughout the milk feeding phase, even in tiny amounts, is crucial for the progressive transition from entire reliance on milk to solid nutrition, which is required to combat the stress produced by nutrient deficiency, digestion inefficiencies, and weight loss after weaning (Khan et al., 2011). Early feed supplementations allow goat kids to be weaned earlier and reach a greater BW at marketing without incurring any penalties (Paez Lama et al., 2014). According to Luo et al. (2000), goat kids’ growth before weaning is essential to their economic value, because birth and weaning weights are related to kid survival and postnatal development.

Dietary intervention is particularly important for the rumen development of goat kids throughout the preweaning period, especially after temporal microbial colonization (Zhang et al., 2019a). The preweaning diet alters rumen microbial structure and VFA production in goats (Abecia et al., 2014). Predominant genera in the non-supplemental goat kids changed from unclassified *Sphingobacteriaceae* to *Prevotella* in the supplementary solid diet goat kids (Lv et al., 2019). In addition, the early starter feed supplementation improved rumen development through the rumen content and the variety and richness of epithelial microbiota in goat kids (Chai et al., 2021). Introducing solid feed to the young calves enhances the rumen microbiota to reach the level of adult ruminants to be more specific and adapted to the new kinds of feeds (Meale et al., 2016).

The pattern and quality of solid feed have a significant impact on the rumen fermentation process (Desnoyers et al., 2011). Without adequate intake of solid food, the rumen may not develop properly, leading to digestive issues and poor nutrient absorption and resistance to diseases and parasites (Ghani et al., 2017). In addition, limited availability, and diversity of feed resources during the preweaning stage can limit gastrointestinal tract microbial development (Belanche et al., 2020b). However, if the solid diet content of fermentation substrates is high, it can provide more VFA to drive rumen growth. Die et al. (2017) stated that the benefits of the solid feed on rumen development are primarily related to its readily fermentable carbohydrates, which can increase VFA production in the rumen. As a result, solid starter diets with high carbohydrate content have been commonly utilized to rear preweaned ruminants (Baldwin et al., 2004; Suarez et al., 2006). The high dietary energy could promote rumen energy production and microbial protein supply through increasing substrate-level and electron transport phosphorylation in the ruminal microbiome, both of which are associated with glucose fermentation (Lu et al., 2019). Therefore, the energy and protein limitation in weaned kid’s had negative effects on the digestion and absorption at the rumen and jejunum levels (Sun et al., 2017). In addition, restricting energy and protein for 6 wk can lower the antioxidant capacity of plasma and the rumen and jejunum epithelium in weaned goat kids and may have a long-term impact (Sun et al., 2012). Therefore, a suitable feed composition is critical for optimal rumen development and core microbiota establishment (Sun et al., 2010; Arshad et al., 2021).

The high-grain diet is a frequent approach for boosting ruminant energy supply (Xu et al., 2018; Cheng et al., 2021). Diets high in grain can boost the formation of VFA, which stimulates the rumen’s growth and absorption activity compared to forage-fed ruminants (Zitnan et al., 1999; Shen et al., 2004). Furthermore, providing goat kids with concentrate may alter the rumen microbiome, improve barrier function, and reduce inflammation (Jiao et al., 2017). Furthermore, the low roughage-high-concentrate (30:70 ratio) diet improved the digestibility of neutral detergent fiber (**NDF**), organic matter, nutrient utilization, rumen fermentation, the proportions of lactic acid, total VFAs, propionate, valerate, the number of *Holotrichs*, *Entodinium*, total protozoa, and bacteria, while the acetate, iso-butyrate, iso-valerate, and anaerobic fungi were lower compared to a high roughage-low concentrate (70:30 ratio) diet in goats (Saini et al., 2012). In addition, the high-grain diet improves the chemosensing of neutral amino acids, fatty acids, and carbohydrates (Tian et al., 2020). Moreover, high-grain diets in goats increased the rumen papillae development and the short-chain fatty acid levels, shifting rumen fermentation toward propionate production (Tian et al., 2020). In addition, the growth of the rumen papillae was greatly accelerated by a medium starch diet containing 35% starch in goats (Zitnan et al., 2003; Odongo et al., 2006; Shen et al., 2020). On the other hand, ruminal acidosis may be produced by a high-grain diet, which may reduce epithelial microbiota alpha diversity than baseline diets in sheep (Seddik et al., 2018) and cattle (Petri et al., 2013). When compared to a 0% grain diet, a 65% grain diet can disturb the ruminal epithelial tight junctions in goats (Liu et al., 2013). Feeding high rumen degradable starch diet to growing goats increased the rumen acetate-to-propionate ratio, reduced rumen papilla development, rumen epithelial VFA absorption, and the capacity to maintain the rumen epithelial homeostasis, thereby decreasing the supply of precursors for hepatic gluconeogenesis and repressing the goats’ performance (Jin et al., 2023). Although the general expectation was that the high-concentrate diets could decrease the acetate-to-propionate ratio in goats (Sun et al., 2010), the authors suggested that impairs animal health by lowering rumen pH and propionate (Jin et al., 2023). Thus, reducing the content of dietary rumen degradable starch enhanced post-ruminal starch digestion and increased plasma glucose, thereby improving amino acid utilization and promoting protein synthesis in the skeletal muscle of goats via the AMPK-mTOR pathway (Liang et al., 2023b). Consequently, feeding ground corn steeped in organic acids such as citric acid successfully reduced the risk of ruminal acidosis by increasing starch resistance to microbial fermentation, leading to a reduction of haptoglobin and tumor necrosis factor concentrations in dairy goats (Shen et al., 2019).

Compared with grain, high-fiber feeds such as hay and forage may mitigate the rumen acidosis problem and abomasum displacement (Klevenhusen et al., 2013; Grilli et al., 2016; Kenny et al., 2018). Forages improve gut fill because forage has poor digestibility in the rumen. Forages expand the ruminal volume, contributing to muscular development (Tamate et al., 1962). In addition, it can encourage rumination and the flow of saliva to the rumen (Hodgson, 2010). Therefore, a reasonable amount of hay for the kids is important to ensure enough consumption of concentrates (Bélanger-Naud, 2020). Thus, it can improve the growth of the goat’s rumen and make sure it reaches its maturity before weaning (De Barbieri et al., 2015) and enhance the health status and decrease respiratory diseases (Bélanger-Naud, 2020). The NDF, which is rich in forage and hay, may have a considerable effect on the rumen VFAs, microorganisms, and microbial proteins, and hence the epithelial-specific transcriptome (Lv et al., 2019; Chai et al., 2021). Without receiving forage during the preweaning stage, the newborns challenged the diet, resulting in reduced forage intake after weaning (Phillips, 2004; Yanez-Ruiz et al., 2015). Thus, creep feed supplementation containing alfalfa enhances the growth performance of goats before weaning (Htoo et al., 2015). In addition, the rumen structural parameters, papillary surface area, and keratinized layer of creep feed with roughage were higher than kids fed only milk (Htoo et al., 2018). In addition, forage can influence the rumen microbial composition. A much larger abundance of the genus *Prevotella* and *Butyrivibrio* was observed in forage-fed goats, while goats fed grains have much larger concentrations of the unclassified species *Bacteroidales* and *Ruminococcaceae* (Grilli et al., 2016). In addition, Zhang et al. (2019c) found that 30 taxa at the genera level were influenced by the diet, 25 enriched taxa in the hay diet group (*Butyrivibrio*, *Pseudobutyrivibrio*, *Fibrobacter*, and unclassified *Christensenellaceae* and Ruminococcaceae, etc.) and 5 enriched taxa (*Selenomonas* 1, *Ruminococcus* and unclassified *Veillonellaceae*) in the high-grain diet group. Moreover, the ruminal microbiota of goats offered diet high hay diet group decreased the relative abundance of *Prevotella*, *Ruminococcus*, *Butyrivibrio*, *Mogibacterium*, and *Moryella* and increased *Clostridium*, *Pseudobutyrivibrio*, *Coprococcus*, and *Desulfovibrio* compared with the grain diet feeding (Zhang et al., 2018b). Despite the abovementioned specific impacts of forages, overfeeding of forages before weaning may have a slight negative effect on kid growth. It reduces the voluntary intake of concentrated feed, resulting in inadequate amounts of VFAs to drive rumen development (Coverdale et al., 2004; Bélanger-Naud, 2020). In calves, performance was impaired when low-energy fibrous feeds were added to texturized coarse calf starters (Hill et al., 2008).

In addition, high-quality roughage feeding should be considered for optimal animal growth. For example, alfalfa is one of the most popular high-quality protein roughages in the world that can promote digestion and absorption of ruminants and improve health status (Sun et al., 2023). Thus, the average daily gain (**ADG**) and ultimate BW of goats fed alfalfa were better than those fed sericea lespedeza (Turner et al., 2005). Alfalfa and timothy may be suitable pasture sources for goats based on results regarding VFA (Ryu et al., 2020). In addition, sugarcane top and king grass diet can modify gene expression patterns in nutrition and energy metabolism and replicate and repair genetic information pathways (Li et al., 2021b). Thus, the abundances of *Firmicutes* and *Euryarchaeota* were higher in goats fed king grass with or without exogenous enzymes (Li et al., 2021b). There are some treatments that could be done to improve roughages, feeding a diet containing 50% anthocyanin-rich black cane silage treated with 0.03% ferrous sulfate and 4% molasses increased total VFAs and *Ruminococcus albus* but decreased rumen ammonia nitrogen (N) and the concentration of methanogenesis in goats (Purba et al., 2023)

Moreover, feeding total mixed rations (**TMR**) is recommended to combine the benefits of forage and concentrated diets and decrease the feed selection (Schingoethe, 2017). The rumen morphological and microbiological development is not only influenced by the chemical composition of the diet but also the diet’s physical form and particle size distribution (Malik et al., 2021). Feeds that are too finely ground or pelleted may not provide enough physical stimulation for rumination, which is important for the development of rumen musculature and function. Total mixed diets with 15% wheat straw pellets (8 mm) improved the development of anatomic papillae and the papillae color compared with the smaller particle size of straw (Malik et al., 2021). Li et al. (2023) found that a rice straw particle size of 4 mm may improve the disappearance rate of nutrients and promote the concentrations of VFAs and improving ruminal relative abundance of *Butyrivibrio* and *Prevotella* compared with other particle sizes (1 and 2 mm). Increasing the particle size of straw from 4 to 8 mm at a 15% inclusion rate in high grain complete pelleted diet resulted in improved DMI with higher ADG and nutrient digestibility, increased rumination rate and rumen pH and lowered the incidence of diarrhea (**Khurshid et al., 2023**). On the other hand, the small particle size of straw in pelleted TMR, can reduce chewing activity, decrease buffering capacity and rumen pH to subacute ruminal acidosis levels (Blanco et al., 2015). Overall, there is still a need for deeper knowledge on the impact of chemical composition and physical characteristics of feeds and diets on neonatal gut development together with long-term productivity trials using kids.

### Volatile Fatty Acids

The VFAs as shown in Table 1 can be employed as signaling molecules, intermediary mediators, and energy sources in hosts (Koh et al., 2016; Shen et al., 2017). The VFA generated in the rumen satisfies 70% to 80% of the energy needs of the rumen epithelium and 50% to 70% of the body’s energy needs (Yeoman and White, 2014; Shen et al., 2017). For the proper development of the ruminal epithelial layer, there has to be a healthy ruminal fermentation that produces short-chain fatty acids in the rumen lumen (Baldwin et al., 2004; Lesmeister et al., 2004; Khan et al., 2011). Thus, the VFAs are chemical stimuli for rumen papillae development and absorption activity (Bergman, 1990; Krause and Oetzel, 2006; Bretschger et al., 2010).

**Table 1.** Major characteristics of rumen volatile fatty acids (VFAs)

Items	Butyrate	Acetate	Propionate
Chemical formula	C_4_H_7_O_2_	C_2_H_3_O_2_	C_3_H_5_O_2_
Production	Diets high in fiber	Diets high in fiber	Diets high in starch and sugar
Function	Ketogenesis	Fatty acid synthesis	Gluconeogenic
Production, %	10% to 20% of total VFAs	50% to 70% of total VFAs	15% to 40% of total VFAs
Health benefits	Anti-inflammatory and gut barrier enhancing effects.	Appetite regulation and the synthesis of milk fat	Anti-inflammatory effects and improve insulin sensitivity

Propionate and butyrate have a stronger stimulatory impact on rumen papillae growth than acetate (Tamate et al., 1962; Bergman, 1990; Suarez et al., 2006). The infusion of sodium acetate at 0.8 g/kg BW increased rumen index and short-chain fatty acids production and absorption, and antioxidant capacity and modulated rumen bacteria in goats (Zhen et al., 2023). In addition, a positive relationship were found between propionate and the formation of rumen papillae in lambs (Herath et al., 2021). Thus, calcium propionate supplementation can enhance the rumen epithelial growth (Baldwin and Connor, 2017; Zhang et al., 2020a). In goats, sodium propionate infusion at 0.8 g/kg BW enhanced total antioxidant capacity and increased the abundance of specific rumen bacteria, such as *Christensenellaceae* R-7, *Butyrivibrio*, and *Rikenellaceae* RC9 gut (Zhen et al., 2023). Butyrate, rather than propionate, is thought to be the initial activator of epithelial length and function (Cheng et al., 2021). This explains the substantial positive associations seen in lambs between rumen papillae height at the dorsal rumen site and butyrate content of rumen fluid (Herath et al., 2021). Moreover, increasing amounts of butyrate (54 to 326 g) lowers the rate of cell death (Mentschel et al., 2001). In addition, butyrate influences mucous membrane resistance, gastrointestinal integrity, and colon health (Szyndler-Nędza et al., 2020). Butyrate can efficiently control histone deacetylation, impact gene expression, and mediate numerous ion transport channels in goat kids (Zhuang et al., 2023b). Increased levels of cyclin D1, cyclin-dependent kinase 4, are associated with intraluminal butyrate infusions’ proliferative effects on the ruminal epithelium and are likely related to goats’ increased absorption of short-chain fatty acids (Malhi et al., 2013). Furthermore, butyrate infusions in the rumen at 0.3 g/kg BW boosted rumen papillae, butyrate levels, and the absorption efficiency of the short-chain fatty acid in goats (Malhi et al., 2013). In addition, the exogenous supplementation of sodium butyrate at 0.5 g/kg BW increased the width of rumen papilla and the thickness of the stratum basale and improved the antioxidant capacity and barrier function in goats (Zhen et al., 2023). Thus, milk replacer supplemented with 0.3% sodium butyrate can be a beneficial ingredient to increase daily growth and antioxidant status in kids during the suckling and weaning periods (Mohamed et al., 2020). Butyrate is also utilized to enhance ruminal barrier formation in weaning calves; however, excessive butyrate in the fully matured rumen of adult cows can cause hyperkeratosis, parakeratosis, and epithelial damage (Aschenbach et al., 2019).

The VFAs once absorbed by the rumen epithelial cells, are oxidized to produce ketones, mainly beta-hydroxybutyrate (**BHB**) and a small part of acetoacetate, which aid in energy transfer, transcription of genes, and metabolism regulation (Zhuang et al., 2023a). Thus, the dietary BHB supplementation is supposed to stimulate development of rumen metabolic functions and the energy supply for rumen epithelium. Accordingly, our team found that a reasonable dose of dietary BHB in early weaned goat kids had a good regulating effect on blood total protein level (Abdelsattar et al., 2021), ADG, feed intake, and rumen weight (Abdelsattar et al., 2022a). However, the high BHB doses in the long term can have adverse effects on goat health by increasing oxidative stress (Abdelsattar et al., 2022a). Zhuang et al. (2023a) revealed that BHB promoted rumen development by altering the gene ontology pathway of nicotinamide adenine dinucleotide dehydrogenase activity and accelerating lipid metabolism. Another study by Chai et al. (2024), showed that dietary BHB improved growth and rumen environment in goat kids through modulation of the structure and composition of the rumen microbiota and enhancement of rumen biosynthesis of VFA.

### Plant Extracts

**Figure 2.**
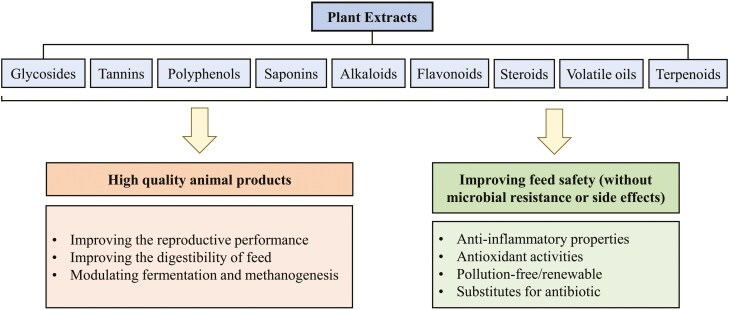
The plant extracts and their health benefits in livestock production.

Several plant extracts can be used as an alternative to antibiotics and to enhance the feed quality, digestibility, as well some of these extracts proved their efficiency in modulating fermentation and methanogenesis resulting in the reduction of methane emissions (Table 2 and Fig. 2). Specific plant extracts containing bioactive compounds and secondary metabolites can have beneficial effects on rumen fermentation and energy utilization and reduce methane and ammonia N production (Busquet et al., 2005), which could be due to their antimicrobial, anti-inflammatory, and alkalizing properties to maintain a stable rumen pH. The supplementation of *Leucaena leucocephala* and *Manihot esculenta* leaves enhanced ammonia N, acetate, and total VFAs production, as well as total protozoa and cellulolytic bacteria populations in the goat’s rumen (Harun et al., 2017). Supplementing mixed grass hay with leaves of *Paraserianthes falcataria*, *Moringa oliefera*, or *Calliandra calothyrsus* enhanced apparent dry matter (**DM**) digestibility, crude protein and crude fiber digestibility, N retention, and rumen propionate while reducing rumen protozoa population (Okoruwa and Ikhimioya, 2020). Dried Greek oregano can increase protease activity and ammonia N production (Paraskevakis, 2018). The antioxidant capacity and anti-inflammatory responses in goat leukocytes may be enhanced by oregano leaves and the production of caproic acid and chemicals related to improved intestinal health (Reyes-Becerril et al., 2021). In addition, *Moringa oleifera* leaf ethanolic extract increased serum antioxidant capacity and modulated the rumen microbiota and metabolites of cashmere goats, it raised the relative abundance of *Treponema* and *Fibrobacter* and reduced the relative abundance of *Prevotella* (Liang et al., 2023a). Moreover, goats fed 6% neem leaf with 15% polyethylene glycol in the concentrate showed improved propionate, *Butyrivibrio fibrisolvens* and *Streptococcus gallolyticus* and reduced methanogens, protozoa, ammonia N, acetate, and butyrate, as well as a lower ratio of acetate to propionate (Taethaisong et al., 2023).

**Table 2.** Effects of various plant extracts in growth performance, health status, and rumen fermentation and microbiota in goats

Plant or plant extract	Dose levels	Supplementation method	Animal	Animals No.	Gender	Age	Body weight	Main findings
Leaves of *Paraserianthes falcataria*, *Moringa oliefera*, or *Calliandra calothyrsus*	100% grass hay with 0% leaf or 70% grass hay with 30% leaf	Added to grass hay	West African dwarf goats	24	Male	8 to 9 mo	8 ± 0.41 kg	Feed intake, BW, N retention, rumen propionate, and apparent digestibility of DM, crude protein, and crude fiber (↑); rumen protozoa population and feed conversion ratio (↓) (Okoruwa and Ikhimioya, 2020).
*Origanum vulgare ssp. Hirtum* whole plant	20 g in the concentrate	Added to the basal diets	Alpine goats	8	Female	4 to 5 yr	50 ± 2.0 kg	Protease activity and ammonia N production (↑); *Peptostreptococcus anaerobius*, *Clostridium sticklandii*, and total methanogen population (↓) (Paraskevakis, 2018).
*Lippia palmeri* Watts leaves	2.6% of DM basis	Added to the basal diets	Anglo-Nubian goats	12	Female	3 yr	47 ± 8.48 kg	The production of caproic acid and chemicals related to improved intestinal health (↑) (Reyes-Becerril et al., 2021).
*Moringa oleifera* leaf powder (MOLP) or ethanolic extract (MOLE)	200 mg/kg BW MOLP, 40 mg/kg BW MOLE	Oral treatment	Cashmere goats	18	Male	2 yr	46.3 ± 2.7 kg	Serum antioxidant capacity and the relative abundance of *Treponema* and *Fibrobacter* (↑); the relative abundance of *Prevotella* (↓) (Liang et al., 2023a).
Neem leaf	6% in concentrate with or without polyethylene glycol	Added to the basal diets	Anglo-Nubian Thai native goats	24	Male	NS	20 ± 2.0 kg	Feed intake, BW, nutrient intake, nutrient digestion, ADG, propionate, *B. fibrisolvens*, and *S. gallolyticus* (↑); blood urea N, methanogens, protozoa, ammonia N, acetate, butyrate, acetate/propionate ratio (↓) (Taethaisong et al., 2023).
Seeds, peel, or whole Manila palm extract	5 mL/kg BW	Added to the basal diets	Crossbred goats	16	Half males and half females	1 to 2 yr	20 ± 3.1 kg	Ruminal propionate (↑); methane production and acetate (↓); feed intake, feed conversion ratio, nutrient digestibility, blood urea N, ruminal pH, ammonia N, total VFAs, butyrate, and microbial populations (NS) (Norrapoke et al., 2023).
*Morinda citrifolia* L. fruit extract	4 g/kg DM	Added to the basal diets	Cashmere goats	14	Male	2.5 yr	46.65 ± 3.36 kg	Apparent digestibility of DM and crude protein, DMI, ADG, N utilization efficiency, total VFAs, and *Firmicutes*/*Bacteroides* (↑); ammonia N and protozoa (↓) (Zhang et al., 2023b)
*Aloe vera* extract	2% and 4% of DMI	Added to the basal diets	Crossbreed (Alpine × Beetal) goats	24	Female	NS	37.5 ± 2.7 kg	Ruminal *B. fibrisolvens* population and milk antioxidant status (↑); the ruminal methanogen, protozoa, *Butyrivibrio proteoclasticus,* and *Ruminococcus flavefaciens* (↓) (Banakar et al., 2023).
Natural betaine extracted from sugar beet molasses	4 g/kg DM	Added to the basal diets	Damascus goats	33	Female	22 to 30 mo	37 ± 0.7 kg	Nutrient digestibility, total VFAs, acetate, butyrate, and acetate/propionate ratio (↑); ruminal ammonia N and propionate (↓); ruminal pH (NS) (Ghoneem and El-Tanany, 2023).
Biochanin A produced mainly in leguminous plants	2 and 6 g/d/goat	Added to the basal diets	Saanen goats	30	Female	NS	63.5 ± 5.6 kg	N metabolism, total VFAs, antioxidant capacity, and the rumen cellulolytic bacteria such as *Prevotella* sp. (↑); ruminal ammonia N and the relative abundance of *Verrucomicrobiota* (↓) (Xu et al., 2023).
Bark of *Amoora rohituka*, peel of Punica granatum, seeds of *Dolichos biflorus*, and root of *Asparagus racemosus*	20 g/kg DMI	Added to the basal diets	Beetal goats	14	Female	NS	NS	Apparent digestibility of DM, cell-mediated immune response, and plasma glucose, albumin, and total amino acids (↑) (Shilwant et al., 2023).
Fish oil and thyme essence, or their mixture	0.2% thyme, 2% fish oil, and a mix (DM concentrate)	Added to the basal diets	Mahabadi goats	28	NS	4 to 5 mo	17.8 ± 2.8 kg	Fish oil: Ether extract digestibility (↑); NDF digestibility and protozoa count (↓) (Ganjkhanlou et al., 2015). Thyme: Ruminal acetate and acetate-to-propionate ratio (↑); ammonia concentration and protozoa count (↓) (Ganjkhanlou et al., 2015).
Fish oil as a replacement of sunflower oil	Sunflower oil (25 g/kg DM) replaced with fish oil at 5, 10, and 15 g/kg	Added to the basal diets	Saanen goats	4	Male	9 mo	20.13 ± 0.25 kg	The intake of eicosapentaenoic acid and docosahexaenoic acid (↑); ruminal ammonia N, total VFAs, acetate, propionate, protozoa population (↓); feed intake, nutrient digestibility, N retention (NS) (Thanh et al., 2018).
Palm, soybean or fish oils	2% of DM	Added to the basal diets	Mahabadi goat kids	24	Male	5 mo	19.4 ± 1.2 kg	Feed intake and growth performance (NS) (Najafi et al., 2012).
Corn oil	30 g/kg DM	Added to the basal diets	Liuyang Black goats	6	Female	12 ± 0.5 mo	19 ± 1.2 kg	Rumen acetate, acetate/propionate ratio, and rumen *biohydrogenation* bacteria, and the genus *Butyrivibrio* 2 (↑); propionate, methane emissions, total unsaturated fatty acids (↓); organic matter digestibility and predominant ruminal fibrolytic (NS) (Zhang et al., 2019d).
Coconut oil	4, 6, and 8 g/d/goat	Sprayed into the back of the kids’ mouth	Hainan Black goats	24	NS	10 d	2.05 ± 0.16 kg	Digestibility of ether extract, ADG, ruminal pH, and serum triglycerides and growth hormone (↑); feed conversion ratio, methane emission, ammonia N, total VFAs, acetate, ruminal microbial enzyme activity, protozoa, and cellulolytic bacteria (↓) (Shi et al., 2020).

DM, dry matter; DMI, dry matter intake; ADG, average daily gain; BW body weight; N, nitrogen ↑, increase; ↓, decrease; ND, no differences.

Furthermore, the supplementation with Manila palm whole extract at a rate of 5 ml/kg goat weight resulted in an increase in propionate, a decrease in methane production, and did not affect ruminal pH, ammonia N, total VFAs, butyrate, and microbial populations (Norrapoke et al., 2023). In addition, pomegranate seed pulp supplementation at 5% and 10% as replacement of wheat bran modulated the methanogen and protozoa population and reduced the methane emission and increased the microbial protein (Jaberi Darmiyan et al., 2023). Feeding cashmere goats the diet containing 0.40% polysaccharide-rich noni fruit extract showed increased *Firmicutes*/*Bacteroides*, N utilization efficiency, and VFAs and the apparent digestibility of DM and crude protein, while it decreased ammonia N and protozoa (Zhang et al., 2023b). Dietary supplementation of *Aloe vera* extract at 2% and 4% of DMI enhanced the ruminal *B. fibrisolvens* population in goats (Banakar et al., 2023). The supplementation of natural betaine extracted from sugar beet molasses increased the total VFAs, acetate, butyrate, and acetate-to-propionate ratio, while it deceased the rumen ammonia N and propionate in goats without affecting ruminal pH (Ghoneem and El-Tanany, 2023). Moreover, biochanin A, an isoflavone phytoestrogen produced mainly in leguminous plants improved N metabolism, total VFAs, antioxidant capacity, and the rumen cellulolytic bacteria such as *Prevotella* sp., while it decreased rumen ammonia N content in goats (Xu et al., 2023). In addition, the increasing doses of polyphenolic and saponin‑rich plant extracts increased total VFAs, propionate and degradability of fiber and DM, whereas it decreased methane production, ammonia N, acetate, and acetate-to-propionate ratio in goats (Shilwant et al., 2023). The combined treatment with *Asparagopsis taxiformis* at 2% DM and phenolic compounds such as phloroglucinol at 6 mM dose in vitro system can alleviate methane production, promote acetate and total VFAs production and therefore improve rumen fermentation in goats (Romero et al., 2023).

Additionally, the nutritional energy density of ruminant diets has been boosted by adding oils throughout the past decade. Nevertheless, because fatty acids can change the metabolism of ruminal fluid by suppressing bacteria, dietary lipid composition and quantity should be considered (Huang et al., 2020). Thus, according to Abdelrahman et al. (2023), fish oil has a suppressive effect on ruminal fermentation and some bacterial populations, including *Prevotella* and *Rikenellaceae* RC9. The addition of fish oil and thyme extract to a goat kid’s diet reduced NDF digestibility while increasing ether extract digestibility and rumen acetate concentration (Ganjkhanlou et al., 2015). Replacement of fish oil for sunflower oil resulted in lower ruminal ammonia N, acetate molar proportions, and ruminal protozoa population in goats (Thanh et al., 2018). Thanh et al. (2018) found that the intake of eicosapentaenoic acid and docosahexaenoic acid was improved by fish oil. Besides, soybean oil showed high amount of linolenic acid in the goat kids’ muscles (Najafi et al., 2012), which is necessary for normal growth and development. In addition, corn oil supplementation promoted rumen biohydrogenation by encouraging the development of biohydrogenation bacteria from the *Lachnospiraceae* family and the genus *Butyrivibrio* 2 (Zhang et al., 2019d). Moreover, a concentrated mixture containing 4% palm oil improved postpartum weight gains, milk yield, and the preweaning growth performance in goats (Otaru et al., 2020). In addition, coconut oil supplementation improves ADG and the feed conversion ratio in goat kids, resulting in lower estimated methane emissions (Shi et al., 2020). Tea tree oil could significantly reduce endotoxin-mediated inflammatory responses in goat rumen epithelial cells, indicating its efficacy in the treatment of subacute rumen acidosis (Hu et al., 2021). According to Kandi et al. (2020), saponification of fatty acid with calcium could decrease the deleterious impact of fat sources on rumen fermentation. They found that supplementing the starter diet of high protein content with calcium salt of linseed oil improved growth performance in lambs, which may be related to enhanced N efficiency (Kandi et al., 2020). In summary, it is recommended to optimize the extraction sites of different plant extracts as well as dosing and for efficient rumen digestibility and absorptive function which affects the health status of young goats. In addition, in mature goats, plant extracts have been used to promote a healthy bacterial environment and fermentation processes. Therefore, more targeted research is needed to fully understand their benefits in young goats during the rumen development phase.

### Probiotics

Eliminating antibiotics from ruminants has resulted in a slew of issues, including growth retardation, poor nutritional utilization, pathogen colonization, dysbiosis, and food safety concerns (Reuben et al., 2022). Antibiotic alternatives such as probiotics or direct-fed microbial have been recommended for improving animal output and protection against numerous infectious, such as diarrhea (Zhang et al., 2021a; Reuben et al., 2022). In the case of goat kids, probiotics can help maintain a diverse and balanced population of beneficial microbes in the rumen, which can improve the efficiency of feed digestion, nutrient absorption, and overall health and productivity of the animal (Table 3). Specific probiotic strains, including *Lactobacillus*, *Bifidobacterium*, *Streptococcus*, *Enterococcus*, and *Propionibacterium* spp., have been effectively employed to boost juvenile health, development, and maturation in ruminants, as reviewed by Reuben et al. (2022). These probiotic strains can also help prevent the overgrowth of harmful pathogens in the gut, reducing the risk of digestive upsets and maintaining overall gut health. The use of yeast products like yeast culture and live yeast cultures (*Saccharomyces cerevisiae*, *Aspergillus oryzae*) are commonly used in ruminant functional feed as a rumen fermentation modulator, stimulant, or performance enhancer which provides a stable environment for rumen fermentation by increasing the pH value and increasing rumen microbial richness and diversity (Özsoy et al., 2013; Yuan et al., 2023). Thus, proper nutrition and management practices, including the use of probiotics, are essential for ensuring the well-being and productivity of early weaned goats. In the context of early weaned goats, newborn ruminants are susceptible to digestive disorders, decreased growth rates, and infections due to the transition from a diet of milk to solid feed. To support the development of a healthy rumen microbiota in early weaned goats, specific probiotic strains can be incorporated into their feed as part of a functional feed formulation.

**Table 3.** Effects of probiotics on growth performance and rumen fermentation and microbiota in goats

Probiotics	Dose levels	Supplement method	Animal	Gender	Animals No.	Age	Body weight	Main findings
Yeast (SC)	2.5 and 5 g/d	Adding into basal diet	Nubian goats	Male	20	3 mo	10 kg	Digestibility of NDF, feed intake, and ADG (↑); ruminal pH, ammonia N, and VFA levels (ND) (Elseed and Abusamra, 2007).
Yeast (SC)	2 × 10^5^, 2 × 10^6^, and 2 × 10^7^ cells/mL in medium	In vitro.	NS	NS	NS	NS	NS	Fungal growth and activity, cellulose degradation and hydrogen, formate, lactate, and acetate production (↑) (Chaucheyras et al., 1995).
Yeast (SC)	3 and 25 g/d	Administration directly to the rumen via fistula	Goats	Female	3	NS	34 ± 4.4 kg	*Diplodinium* and protozoa count, fibrolytic enzyme activities, the disappearance of cell wall carbohydrates, total VFAs, acetate, butyrate, and acetate/propionate ratio (↑); propionate (↓); the disappearance of DM and ADF (ND) (Kowalik et al., 2011).
Yeast (SC)	1.5%, 3%, and 4.5% on fed basis	Added as concentrates	Saanen × Sami goats	Male	48	3.5 to 4 mo	15.6 ± 0.7 kg	Weight gain and rumen ammonia N (↑); ruminal VFA and plasma total protein, albumin, urea N, total cholesterol, triglyceride, and the activities of ALT and AST (ND); the coliform bacteria in the rumen and feces (↓) (Özsoy et al., 2013).
Yeast (SC)	3 kg/ton concentrate	Added to the basal diet	Balady goats	Male	12	12 mo	NS	Total VFAs, ruminal protozoa, and papillae length, width and density (↑); ammonia N (↓) (Abd-Elkader et al., 2019).
Yeast (SC)	0.30%, 0.60%, and 1.20% of basal diet	Added to the basal diet	Macheng Black × Boer goats	Female	12	5.0 ± 1.0 mo	17 to 19 kg	The dose of 0.60%: rumen pH, acetate/propionate ratio, ammonia N, total VFAs, acetate, propionate, butyrate, and the degradability of DM, NDF, and ADF (↑) (Xue et al., 2022b).
Yeast (SC)	3.0 and 4.5 g/d/goat	Added to the basal diet	Guanzhong goats	Female	60	4.0 ± 0.50 mo	19.65 ± 0.41 kg	ADG, DMI, ruminal cellulolytic bacteria abundance and enzyme activity, N utilization, and immune status (Zhang et al., 2023a).
CB, SC, and their mixture	0.05% CB, 0.60% SC, and a mix (0.05% CB and 0.60% SC)	Added to the basal diet	Macheng Black × Boer goats	Male	48	5.0 ± 1.0 mo	22.25 ± 4.26 kg	The ruminal pH, cellulolytic enzymes, ammonia N, total VFAs, propionate, butyrate, ADG, and the digestibility of DM, NDF, and ADF (↑) (Xue et al., 2022a).
CB, SC, and their mixture	0.05% CB, 0.60% SC, and a mix (0.05% CB and 0.60% SC)	Added to the basal diet	Macheng black × Boer goats	Male	12	6.0 ± 1.0 mo	20.21 ± 2.30 kg	Rumen pH, rumen cellulolytic enzymes, total VFAs, acetate, and propionate, DMI, ADG, and the digestibility of DM, NDF, and ADF (↑); the ammonia N only in CB group (↑) (Cai et al., 2021).
CB, SC, and their mixture	0.30% SC plus 0.05% CB, 0.30% SC plus 0.10% CB, 0.60% SC plus 0.05% CB, and 0.60% SC plus 0.10% CB	Added to the basal diet	Macheng Black × Boer goats	Half males and half females	30	5.0 ± 1.0 mo	19 to 21 kg	Rumen pH, ruminal cellulolytic enzymes, ammonia N, acetate, propionate, total VFAs, vitamins B1 and B2, niacin, DMI, ADG, and the digestibility of DM, NDF, and ADF (↑) (Cai et al., 2023).
Selenium yeast	0.3 mg/kg diet	Added to the basal diet	Cross breed goats	Male	10	4 mo	10.5 kg	Blood selenium, glutathione peroxidase activity, propionate and SCFA in the colon, colon weight, and the colonic histological parameters (↑); pH of colonic digesta (↓) (Abbasi et al., 2018).
Selenium yeast	0.3 mg/kg diet	Added to the basal diet	Cross breed goats	NS	10	110 to 130 d	9.93 to 10.71 kg	The propionate and SCFA, the height, width, and density of rumen papillae, rumen epithelium thickness, the glutathione peroxidase activity (↑); rumen pH (↓) (Shahid et al., 2020).
Selenium yeast	2.4 and 4.8 mg/kg	Added to the basal diet	Qianbei-pockmarked weather goats	NS	18	NS	25.75 ± 1.75 kg	High dose: levels of butyrate and valerate (↑). Low dose: the ruminal propionate, iso-butyrate, iso-valerate, and caproic acid, the abundance of *Clostridium*, carbohydrate metabolism pathways (↑); the abundances of *Euryarchaeota*, *Proteobacteria*, *Methanobrevibacter*, and *Sarcina* (↓) (Tian et al., 2022).
*Bacillus amyloliquefaciens* 9	0.3% (*w/v*)	Added to the raw milk	Saanen goats	Female	12	30 ± 2 d	6.5 ± 1.05 kg	Serum immunoglobulin G, interleukin 4, and interleukin 6 (↑); diarrhea (↓); fecal microbiota (ND) (Zhang et al., 2021a).
*Streptococcus caviae* RM296 (SRB4) and sulfur (S)	Control, 0.5 ml SRB4/kg BW, 0.095% S DMI, 0.095% S plus 0.5 ml SRB4, 0.19% S, 0.19% S plus 0.5 mL SRB4	Added to the basal diet	Goats	Male	36	5 to 6 mo	10.08 ± 0.21 kg	Methane (↓); feed intake, ADG, feed conversion ratio, and nutrient digestibility (ND) (Uniyal et al., 2022).

DM, dry matter; DMI, dry matter intake; ADG, average daily gain; VFA, Volatile fatty acid; SCFA, short-chain fatty acids; NDF, neutral detergent fiber; ADF, acid detergent fiber; SC, *Saccharomyces cerevisiae*; CB, *Clostridium butyricum*; ALT, alanine amino transferase; AST, aspartate amino transferase; N, nitrogen; ↑, increase; ↓, decrease; ND, no differences.

The addition of yeast (*Saccharomyces cerevisiae*) to goat kids fed forage sorghum hay enhanced NDF digestibility, feed intake, and ADG (Elseed and Abusamra, 2007). The ruminal pH, ammonia N, and VFA levels showed numerical increase with yeast supplementation (Elseed and Abusamra, 2007). In young ruminants during weaning, yeast improved the growth of cellulolytic bacteria and lactate-utilizing bacteria, resulting in higher feed intake, growth performance, and rumen stability leading to a diarrhea reduction (Chaucheyras et al., 1995). Adding yeast metabolites (*Saccharomyces cerevisiae*) to the diet enhanced the overall number of protozoa, fibrolytic enzyme activities, rumen liquid acidity, the disappearance of cell wall carbohydrates, and the molar percentage of acetate and butyrate (Kowalik et al., 2011). Dietary live yeast culture improves weight gain and rumen ammonia N levels in goats and mesophilic bacteria while reducing the coliform bacteria in the rumen and feces, indicating a positive impact on animal health (Özsoy et al., 2013). In addition, the live yeast strain of *Saccharomyces cerevisiae* increased total the ruminal total VFAs, protozoal count, papillae length, width and density, but decreased the ruminal ammonia N concentration of Balady goats (Abd-Elkader et al., 2019). Moreover, *Saccharomyces cerevisiae* (0.60% of the basal diet) improved the rumen pH, acetate-to-propionate ratio, the concentrations of ammonia N, total VFAs, acetate, propionate, butyrate, and the degradability of DM, NDF, and acid detergent fiber (**ADF**) in goats (Xue et al., 2022b). In addition, the active dry yeast containing *Saccharomyces cerevisiae* improved ADG, DMI, ruminal cellulolytic bacteria abundance and enzyme activity, N utilization, and immune status of dairy goats (Zhang et al., 2023a). Furthermore, *Clostridium butyricum*, *Saccharomyces cerevisiae*, and their mixture improved ruminal pH, the activities of cellulolytic enzymes, the concentrations of ammonia N, total VFAs, propionate, butyrate, ADG, and the nutrients digestibility in goats (Cai et al., 2021, 2023; Xue et al., 2022a).

Furthermore, the supplementation of selenium yeast, a vital resource of synthetic organic selenium, has been shown to change the microbial count and increase the pace of fermentation in the rumens of sheep and goat (Abbasi et al., 2018). Selenium yeast increased propionate and total short-chain fatty acid contents and lowered pH in the ruminal fluid of goats (Shahid et al., 2020). In addition, the height, width, and density of rumen papillae, as well as the rumen epithelium thickness and glutathione peroxidase activity increased in Selenium yeast-treated goats (Shahid et al., 2020). Moreover, selenium yeast (2.4 mg/kg) improved the rumen fermentation parameters including propionate, the carbohydrate metabolism pathways, and upregulating glycosyl transferase and carbohydrate-binding module pathways in Qianbei-pockmarked weather goats (Tian et al., 2022). Furthermore, *Bacillus amyloliquefaciens* 9 can be used to treat and prevent goat kid diarrhea by upregulating the concentrations of immunoglobulins and interleukins (Zhang et al., 2021a). This suggests that *Bacillus amyloliquefaciens* 9 improves intestinal health by altering microbial homeostasis (Zhang et al., 2021a). Moreover, feeding of sulfate‑reducing bacteria (*Streptococcus caviae* RM296) as a microbial feed additive along with sulfur (as sodium sulfate) is capable of reducing enteric methane emission without any adverse effect on rumen fermentation and digestibility of the nutrients in goats (Uniyal et al., 2022). Further investigations are still needed to confirm the positive effects of these strains on modulating the gastrointestinal tract microbiota and alleviating weaning stress.

## Microbiota Transplantation

Rumen contents collected from alive or slaughtered animals have high nutritional value, health benefits, and economic value, providing an environmentally friendly solution for waste management. Rumen microbiota transplantation (**RMT**) involves inoculating the complex microbial community with a wide range of rumen microorganisms, including bacteria, protozoa, fungi, and archaea from the rumen of an adult goat to a young goat to help establish a healthy and diverse gut microbiome and alleviate weaning stress. The simpler and less diversified gastrointestinal microbiota of kids responds to exogenous inoculation better than that of adult animals due to its reduced colonization resistance (Yanez-Ruiz et al., 2015). Accordingly, it is of importance to use a RMT technique to counteract the poor colonization that occurs under artificial milk feeding (Kim et al., 2021). In addition, this technology has the potential to improve rumen development, nutrient absorption, and overall health in young goats, leading to improved growth rates and feed efficiency. It may also have implications for enhancing rumen function in other ruminant species and could contribute to sustainable livestock production.

Belanche et al. (2020a) showed that the oral inoculation of fresh rumen fluid from adult goats enriched early rumen protozoa colonization and rumen VFA synthesis and absorption, facilitating a smooth transition from milk to solid feed and allowing for the implementation of early weaning strategies in newborn goat kids. A similar study by Palma-Hidalgo et al. (2021), revealed more complex and diverse bacterial, methanogenic, protozoal, and fungal communities with the inoculation of rumen fluid from adult goats to young goats. In newborn lambs, the inoculation of rumen contents of ewes can promote their ruminal microbiota communities (De Barbieri et al., 2015; Yu et al., 2020). In addition, the inoculation with adult goat ruminal fluid had a beneficial impact on growth, health and metabolism in newborn lambs through modulating the gut microbial structure by increasing the relative abundance of *Rikenellaceae*_RC9 and decreasing the relative abundance of *Akkermansia* and *Escherichia-Shigella* (Fu et al., 2023). A recent study by Zhao et al. (2023) showed that both rumen solid and fluid contents from adult sheep could efficiently modulate and enhance the in vitro fermentation efficiency and microbiota structure.

Not only minimizing the weaning stress, rumen transplantation has been also used to treat animals exposed to botanical toxins (DePeters and George, 2014) and accelerate rumen homeostasis recovery in sheep and cows suffering from subacute ruminal acidosis (Liu et al., 2019); Mu et al. (2021). In addition, the exchange of ruminal contents between high- and low-efficiency animals could alter the rumen bacterial community and improve the performance (Weimer et al., 2017). In addition, the RMT from goats to mice improved the colonization of fiber-degrading bacteria leading to high content of butyrate for protecting the colonic epithelial barrier and stimulating energy metabolism (Chen et al., 2022). Furthermore, intestinal microbiota transplantation restores the gut microbiota in bacterial-restricted C57/6J mice, therefore reducing inflammation and enhancing mucosal barrier function (Zhang et al., 2022). Fecal microbiota transplantation in piglets can help avoid animal diarrhea by introducing certain bacteria to the recipients (Hu et al., 2018). Moreover, fecal microbiota can be also used for ameliorating diarrhea in preweaning calves with alterations in their gut microbiota and promoting subsequent body mass gain during the fattening stage (Kim et al., 2021). Recently, the washed microbiota has been used instead of the weight of stool to reduce the adverse events of fecal microbiota transplantation (Zhang et al., 2020b). Additional research is still needed to clearly identify beneficial microorganisms in the rumen or feces and the selection criteria of donors for promoting the health status and the productive efficiency.

## Future Perspectives

The early stage of life is important to manipulate rumen development in young ruminants using several feeds and feed additives instead of using antibiotics. Numerous studies are being conducted annually for optimizing reliable functional feed formulations and strategies to be used in practice for better early rumen development and digestibility of feeds to support the growth performance, production efficiency, and health status of young goats. For example, the exogenous VFA and inoculation with rumen fluid in early life have recognized effects in improving the rumen morphology and metabolic function and the rumen microbial structure. However, other feed additives and feeding strategies could have limited effects or severe effects on the rumen function, such as oils under wrong inclusion rates, as well as the contamination with specific microorganism during RMT. The efficacy of feed additives in the experimental animals could be restricted to several conditions such as nutrition, composition of feed, feeding management, husbandry, environment, and animal breed and age within the experiment. Weaning age and method and the incidence of some diseases during weaning such as diarrhea can be counted, making it challenging to predict consistent results. Thus, the applicability and efficacy of these feed strategies in the large-scale commercial farms require further investigations to know when and how to use them. In addition, studies should also cover the gaps of knowledge about the long-term effects of these feeding systems on the productivity of goats, economic evaluations, accurate inclusion rates, mode of action, and their safety on goats and goats’ products. Currently, the feed industry can benefit from several new technologies that can affect the characteristics of feed and/or the digestion and performance in animals which require further investigations.

## Conclusions

There is increasing demand for sustainable and eco-friendly agricultural practices that have unique characteristics to promote the growth and maturation of the rumen and contribute to the goal of reducing environmental impact of early weaning. In this context, several liquid and solid feeds as well as feed additives typically include ingredients such as prebiotics, probiotics, enzymes, and plant extracts that support the development of a healthy rumen environment. Furthermore, the emerging technology regarding the inoculation of rumen microbial contents from the adult goats to younger goats or from the healthy animals to the unhealthy was covered in establishing a healthy rumen microbiota. These up-to-date findings have a significant role in understanding the function of feeds and the interactions between the feed, microbiome, and the goat’s performance.

The early solid diets for the preweaned goats can provide more VFA and modulate the digestive tract microbiota to drive the development of rumen epithelium and the rumen absorptive function, reducing the goat’s full dependence on milk and the stress of weaning transitions. However, abrupt solid diet during the sudden weaning could attenuate gut injury and decrease the social interactions between the young kids and their dams. Plant extracts could modulate the rumen microbiota and metabolites, depending on the type and dose of administration, leading to the reduction of methane and ammonia N production and the improvement of rumen fermentation, nutrient digestibility, immunity, and antioxidant effects. However, it is important to carefully consider the potential risks of toxicity and palatability issues and to ensure the safe dosage and the effective use of plant extracts in small ruminants. Probiotics and rumen microbial transplants could enhance the gut microbiota of the early weaned goats and can thus reduce gut inflammation and enhance the integrity of mucosal barrier and growth performance. Yet, the lack of standardization of strain and dosage, high costs, resistant development to specific strains over time, interactions with medications or antibiotics, and contamination risks must be considered. Proper selection, administration, and monitoring of probiotic supplements and microbial transplants are crucial to maximize their benefits and ensure the health and well-being of young goats during the weaning process.
